# *Francisella novicida* Mutant XWK4 Triggers Robust Inflammasome Activation Favoring Infection

**DOI:** 10.3389/fcell.2021.743335

**Published:** 2021-11-18

**Authors:** Yu Guo, Rudi Mao, Qingqing Xie, Xiaojie Cheng, Tao Xu, Xiaoyuan Wang, Yan Du, Xiaopeng Qi

**Affiliations:** ^1^School of Life Sciences, University of Science and Technology of China, Hefei, China; ^2^Key Laboratory of Animal Models and Human Disease Mechanisms of the Chinese Academy of Sciences/Key Laboratory of Bioactive Peptides of Yunnan Province, Kunming Institute of Zoology, Chinese Academy of Sciences, Kunming, China; ^3^Key Laboratory for Experimental Teratology of the Ministry of Education, Advanced Medical Research Institute, Cheeloo College of Medicine, Shandong University, Jinan, China; ^4^State Key Laboratory of Food Science and Technology, Jiangnan University, Wuxi, China; ^5^Department of Clinical Laboratory, The First Affiliated Hospital of Kunming Medical University, Kunming, China; ^6^Yunnan Key Laboratory of Laboratory Medicine, Kunming, China; ^7^Department of Clinical Laboratory, Qilu Hospital of Shandong University, Jinan, China

**Keywords:** *Francisella novicida*, XWK4, AIM2, NLRP3, ASC

## Abstract

Bacterial infection tendentiously triggers inflammasome activation, whereas the roles of inflammasome activation in host defense against diverse infections remain unclear. Here, we identified that an ASC-dependent inflammasome activation played opposite roles in host defense against *Francisella novicida* wild-type (WT) U112 and mutant strain XWK4. Comparing with U112, XWK4 infection induced robust cytokine production, ASC-dependent inflammasome activation, and pyroptosis. Both AIM2 and NLRP3 were involved and played independent roles in XWK4-induced inflammasome activation. Type II interferon was partially required for XWK4-triggered inflammasome activation, which was different from type I interferon dependency in U112-induced inflammasome activation. Distinct from *F. novicida* U112 and *Acinetobacter baumannii* infection, *Asc^–/–^* mice were more resistant than WT mice response to XWK4 infection by limiting bacterial burden *in vivo*. The excessive inflammasome activation triggered by XWK4 infection caused dramatical cell death and pathological damage. Our study offers novel insights into mechanisms of inflammasome activation in host defense and provides potential therapeutic approach against bacterial infections and inflammatory diseases.

## Introduction

*Francisella tularensis* is an extremely virulent and facultative intracellular bacterial pathogen capable of causing human disease called tularemia (Oyston et al., 2004). *Francisella novicida* (*F. novicida*) is the least virulent subspecies that causes severe disease in mice but not in immunocompetent humans and served as a mouse strain to study *Francisella* pathogenesis (Weiss et al., 2007). Increasing evidences indicate that *Francisella* employs a number of virulence factors for evading host immune responses to establish high pathogenicity through entering host cells, escaping from phagosome, and replicating within the cytosol (Brotcke et al., 2006; Qin et al., 2009; Rasmussen et al., 2014; Bachert et al., 2019). The important transcription factor MglA regulates the expression of many genes located in the *Francisella* pathogenicity island (FPI) and is required for both *Francisella* virulence *in vivo* and cytosol replication (Mariathasan et al., 2005; Weiss et al., 2007). Instead, certain virulence factors such as FTT0584 are remarkable for *Francisella* pathogenesis through suppressing cell death without affecting *Francisella* intracellular replication (Weiss et al., 2007). Lipopolysaccharide (LPS) is a key virulence factor of *Francisella*, which is composed of lipid A, core oligosaccharide, and O-antigen. *Francisella* LPS is inert and essential for evading host immune responses due to its lack of interaction with Toll-like receptor 4 (TLR4) (Gunn and Ernst, 2007). Mutants defective in the biosynthesis of lipid A and core oligosaccharide and ligation of O-antigen to LPS core oligosaccharides have been demonstrated to be significantly attenuated in the mouse models of tularemia for both *F. tularensis* and *F. novicida* subspecies. Interestingly, all these mutants have high efficacy to induce robust inflammatory responses (Wang et al., 2007; Rasmussen et al., 2014), indicating that most virulence factors of *Francisella* contribute to the immune evasion process.

Inflammasome is a large cytoplasmic multiprotein complex that mediates pyroptotic cell death and subsequent secretion of proinflammatory cytokines interleukin (IL)-1β and IL-18 in response to microbial infection and cellular damage (Xu et al., 2019). One of the most remarkable advances in the immune responses to *Francisella* infection during the last decade is the observation of mechanisms by which *Francisella* triggers AIM2 inflammasome activation. Initially, the Monack group demonstrated that caspase-1- and ASC-mediated cell death and release of the proinflammatory cytokines IL-1β and IL-18 were essential for host defense against *F. novicida* infection (Mariathasan et al., 2005). They also reported that type I interferon (IFN-I) signaling was required for *Francisella*-induced inflammasome activation, although the engaged inflammasome receptor was not known yet (Henry et al., 2007). Later on, cytosolic DNA-activated AIM2 inflammasome was shown to be required for *F. novicida* infection-induced caspase-1 activation and pyroptosis (Fernandes-Alnemri et al., 2010; Jones et al., 2010). Recently, IFN-I-activated transcription factor IRF1, GBP proteins, and IRGB10 were defined as critical regulators for *F. novicida* intracellular bacteriolysis, cytosolic DNA release for AIM2 engagement, and subsequent inflammasome activation (Man et al., 2015, 2016; Meunier et al., 2015). However, type I IFN signaling and AIM2 inflammasome play opposite roles in host defense against *F. novicida* infection, which might attribute to the additional functions of IFN-I in the other cell death pathways including necroptosis and apoptosis (Jones et al., 2010; Li F. et al., 2018; Li Y. et al., 2018; Zhu et al., 2018; Guo Y. et al., 2020). Therefore, the detailed mechanisms underlying the interplay between cell death and inflammation and how they contribute to the host defense during *Francisella* infection need to be further examined.

Previous investigations showed lipid A molecules of LPS in *F. novicida* lacking the 4′-monophosphate group, which is removed by the lipid A 4′-phosphotase (LpxF) (Wang et al., 2006). The 4′-phosphatase deleted strain of *F. novicida* (XWK4) retains the 4′-phosphate group and 3′-hydroxyacyl chain on the diglucosamine backbone of lipid A (Wang et al., 2006; Gunn and Ernst, 2007). XWK4 mutant is demonstrated as avirulent through triggering robust inflammatory responses and defective replication within macrophage (Wang et al., 2007; Kanistanon et al., 2012). Cell death-driven inflammation is an intrinsic immune defense mechanism during bacterial infection (Peltzer and Walczak, 2019; Priem et al., 2020). Despite the significant advances in the regulation of TLR4 signaling by distinct lipid A molecules, the interplay between lipid A molecule of *F. novicida* and host cell death pathway in particular the inflammatory cell death-pyroptosis and inflammasome activation remains unclear. In this study, we identified that *F. novicida* mutant XWK4 dramatically induced robust cytokine production and pyroptosis that was dependent on both AIM2 and NLRP3 inflammasomes. Interestingly, the inflammasome activation negatively regulated host defense against XWK4 infection due to excessive pathological damage. Our study not only provides novel insights into the roles of inflammasome activation in host defense mechanism but also promotes the development of intervention strategies against *Francisella* infection.

## Results

### *Francisella novicida* Mutant XWK4 Triggers More Inflammatory Cytokine Production and Cell Death Than Wild-Type (WT) U112 Strain

**FIGURE 1:**
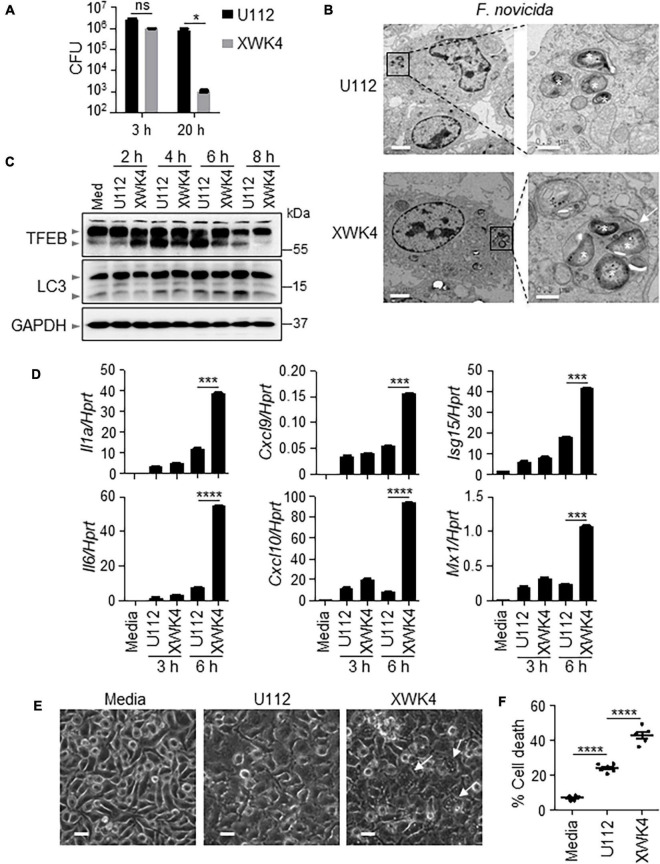
*F. novicida* mutant strain XWK4 enhances inflammatory cytokines expression and cell death. **(A)** BMDMs from WT mice were infected with *F. novicida* WT strain (U112, 20 MOIs) and mutant strain (XWK4, 20 MOIs) for 3 h, and intracellular bacteria were enumerated at indicated times. **(B)** Transmission EM analysis of U112- and XWK4-infected (100 MOIs) BMDMs for 2 h. Asterisk indicates the individual intracellular bacteria. Arrow indicates the vesicle that contains multiple bacteria. Scale bars: 2 μm for left and 0.5 μm for right. **(C)** Immunoblot analysis of TFEB and LC3 in uninfected and U112- and XWK4-infected (100 MOIs) BMDMs at indicated times. **(D)** Expression of *Il1a*, *Il6*, *Cxcl9*, *Cxcl10*, *Isg15*, and *Mx1* was analyzed by qRT-PCR in U112- and XWK4-infected (100 MOIs) BMDMs for indicated times. **(E,F)** Cell death was analyzed by microscope **(E)** and LDH release assay **(F)** for U112- and XWK4-infected (200 MOIs) BMDMs (14 h). Arrow indicates the dying cell. Scale bars: 20 μm for **(E)**. Each symbol indicates individual sample for **(F)**. *p < 0.05; ***p < 0.001; ****p < 0.0001; ns, not significant (two-sided Student’s *t*-test without multiple comparisons correction). Data are representative of three independent experiments.

Both IFN-I and AIM2 were required for *F. novicida*-triggered inflammasome activation and host defense (Jones et al., 2010; Man et al., 2015). To define whether AIM2 and type I IFN were essential for the attenuated virulence in wild-type (WT) mice during XWK4 infection, we infected WT, *Aim2^–/–^*, and *Ifnar^–/–^* mice with *F. novicida* WT U112 strain and mutant XWK4, separately. Notably, both WT and knockout mice lost body weight and succumbed to the infection with U112 strain but not XWK4 mutant (Supplementary Figure 1). These data indicate that AIM2 signaling and IFN-I signaling are not the key factors for host defense against XWK4 infection. To investigate the immune responses induced by XWK4 mutant, WT bone marrow-derived macrophages (BMDMs) were infected with U112 and XWK4, and bacterial killing activity was assessed. The bacterial entry of XWK4 and U112 was comparable, while intracellular replication of XWK4 was significantly inhibited compared with U112 (Figure 1A). Interestingly, transmission electron microscopy revealed that in contrast to U112, XWK4 tendentiously accumulated in one big vesicle (Figure 1B). To examine whether the autophagy and lysosomal biogenesis pathway were differently regulated by U112 and XWK4, we investigated the expression pattern of TFEB and LC3, which are hallmarks of lysosomal biogenesis and autophagy, respectively. The lysosomal biogenesis and autophagy modulated by XWK4 infection were slightly reduced compared with those by U112 infection (Figure 1C). Strikingly, the expression levels of inflammatory cytokine and IFN-I signaling genes in response to XWK4 infection were significantly higher than those of U112 treatment (Figure 1D). After longer treatment, we found that XWK4 induced much more cell death in Wild-Type BMDMs than in U112 (Figures 1E,F).

### XWK4 Is Able to Trigger Higher Inflammasome Activation Than Wild-Type U112

**FIGURE 2:**
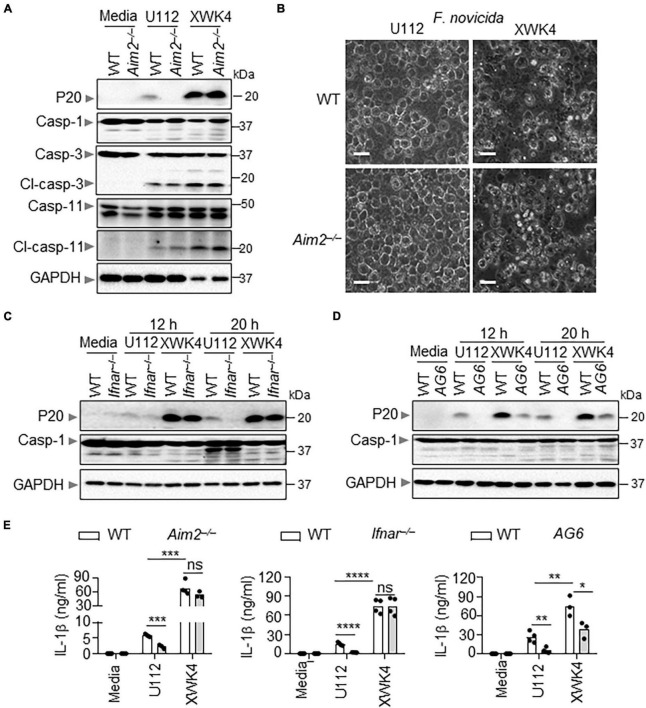
Compared with U112, XWK4 triggers higher intensity of inflammasome activation. **(A)** Immunoblot analysis of caspase-1 (cleaved, P20), caspase-3 (cleaved, Cl-casp-3), and caspase-11 (cleaved, Cl-casp-11) in WT and *Aim2^–/–^* BMDMs with and without infection of U112 and XWK4 (200 MOIs) for 20 h. **(B)** Cell death analysis of WT and *Aim2^–/–^* BMDMs infected with U112 and XWK4 (200 MOIs) for 14 h. Scale bars: 20 μm. **(C)** Immunoblot analysis of caspase-1 (cleaved, P20) in WT and *Ifnar^–/–^* BMDMs with and without infection of U112 and XWK4 (200 MOIs) for indicated times. **(D)** Immunoblot analysis of caspase-1 (cleaved, P20) in WT and *AG6* BMDMs with and without infection of U112 and XWK4 (200 MOIs) for indicated times. **(E)** Analysis of IL-1β release in WT and knockout BMDMs as indicated infected with U112 and XWK4 (200 MOIs) for 20 h. *P < 0.05; **P < 0.01; ***P < 0.001; ****P < 0.0001; ns, not significant(two-sided Student’s *t*-test without multiple comparisons correction). Data are representative of three independent experiments.

To investigate how XWK4 triggers robust cell death, we performed inflammasome activation analysis in control and bacterial pathogen infected WT and knockout BMDMs. Remarkably, we noted that caspase-1 activation was increased in BMDMs infected with XWK4. Furthermore, the caspase-1 activation induced by XWK4 was not dependent on AIM2 that was required for U112-induced inflammasome activation (Figure 2A). In contrast, the activation of caspase-3 and caspase-11 was comparable between U112 and XWK4 treatment (Figure 2A). Consistently, XWK4 induced more cell death than U112 in both WT and *Aim2^–/–^* BMDMs (Figure 2B). To define which pathway was required for XWK4-activated inflammasome activation, we analyzed caspase-1 activation in *Ifnar^–/–^* and *Ifnar^–/–^Ifnγ^–/–^* double knockout (*AG6*) BMDMs in response to XWK4 infection. The caspase-1 activation induced by XWK4 was partially reduced in *AG6* BMDMs but not in *Ifnar^–/–^* BMDMs (Figures 2C,D). In line with the caspase-1 activation data, the IL-1β release induced by XWK4 also was partially reduced in *AG6* BMDMs (Figure 2E). In addition, mTORC1 signaling was not essential for XWK4-induced caspase-1 activation through rapamycin treatment and *Raptor^–/–^* BMDMs (Supplementary Figure 2).

### Both AIM2 and NLRP3 Are Required for XWK4-Induced Inflammasome Activation

**FIGURE 3:**
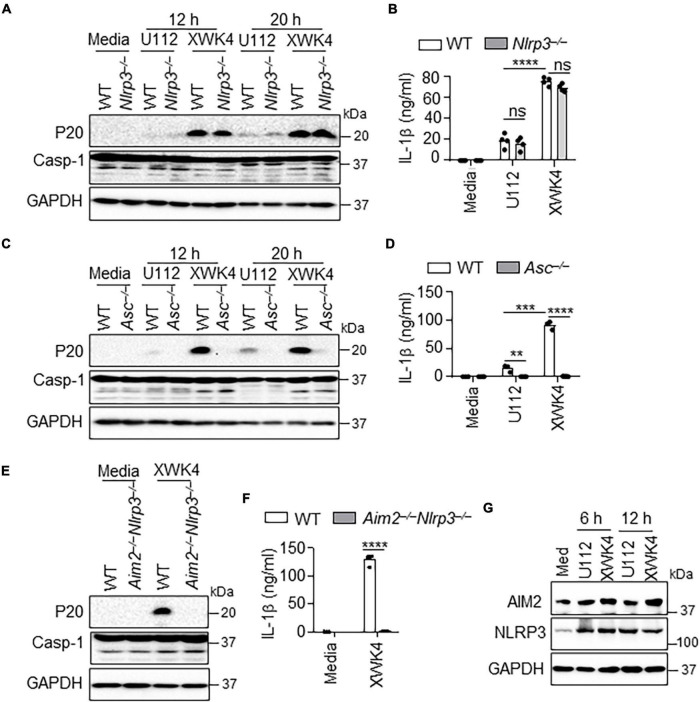
XWK4 triggers both AIM2 and NLRP3 inflammasomes activation. **(A)** Immunoblot analysis of caspase-1 (cleaved, P20) in WT and *Nlrp3^–/–^* BMDMs with and without infection of U112 and XWK4 (200 MOIs) for indicated times. **(B)** Analysis of IL-1β release in WT and *Nlrp3^–/–^* BMDMs with and without infection of U112 and XWK4 (200 MOIs) for 20 h. **(C)** Immunoblot analysis of caspase-1 (cleaved, P20) in WT and *Asc^–/–^* BMDMs with and without infection of U112 and XWK4 (200 MOIs) for indicated times. **(D)** Analysis of IL-1β release in WT and *Asc^–/–^* BMDMs with and without infection of U112 and XWK4 (200 MOIs) for 20 h. **(E,F)** Immunoblot analysis of caspase-1 **(E)** and analysis of IL-1β release **(F)** in WT and *Aim2^–/–^Nlrp3^–/–^* BMDMs with and without infection of U112 and XWK4 (200 MOIs) for 20 h. **(G)** Immunoblot analysis of AIM2 and NLRP3 in WT BMDMs with and without infection of U112 and XWK4 (100 MOIs) for indicated times. **P < 0.01; ***P < 0.001; ****P < 0.0001; ns, not significant(two-sided Student’s *t*-test without multiple comparisons correction). Data are representative of three independent experiments.

NLRP3 was reported to synergistically cooperate with AIM2 for *Listeria monocytogenes* and *Aspergillus* infection-induced inflammasome activation (Kim et al., 2010; Karki et al., 2015). Given that the *F. novicida* XWK4 mutant alters lipid A structure, we postulated that XWK4 might activate NLRP3 inflammasome leading to pyroptosis. Thus, we infected WT and *Nlrp3^–/–^* BMDMs with U112 and XWK4. Of note, the caspase-1 activation and IL-1β production induced by XWK4 were not affected by NLRP3 deletion (Figures 3A,B). As hypothesized, the caspase-1 activation and IL-1β production induced by XWK4 were indeed dependent on the common inflammasome adaptor ASC protein (Figures 3C,D). To test whether XWK4 simultaneously triggered both AIM2 and NLRP3 inflammasome, we infected WT and *Aim2^–/–^Nlrp3^–/–^* double knockout BMDMs with XWK4 and analyzed caspase-1 activation and subsequent IL-1β release. Interestingly, the caspase-1 activation and IL-1β production triggered by XWK4 were abolished in *Aim2^–/–^Nlrp3^–/–^* BMDMs (Figures 3E,F). Furthermore, the expression of NLRP3 and AIM2 was comparably induced by U112 and XWK4 infection (Figure 3G). Taken together, these data indicate that XWK4 engages with both NLRP3 and AIM2 for inflammasome activation and NLRP3 and AIM2 play independent roles in the inflammasome activation during XWK4 infection.

### Excessive Inflammasome Activation Is Deleterious for Host Defense Against XWK4 Infection

**FIGURE 4:**
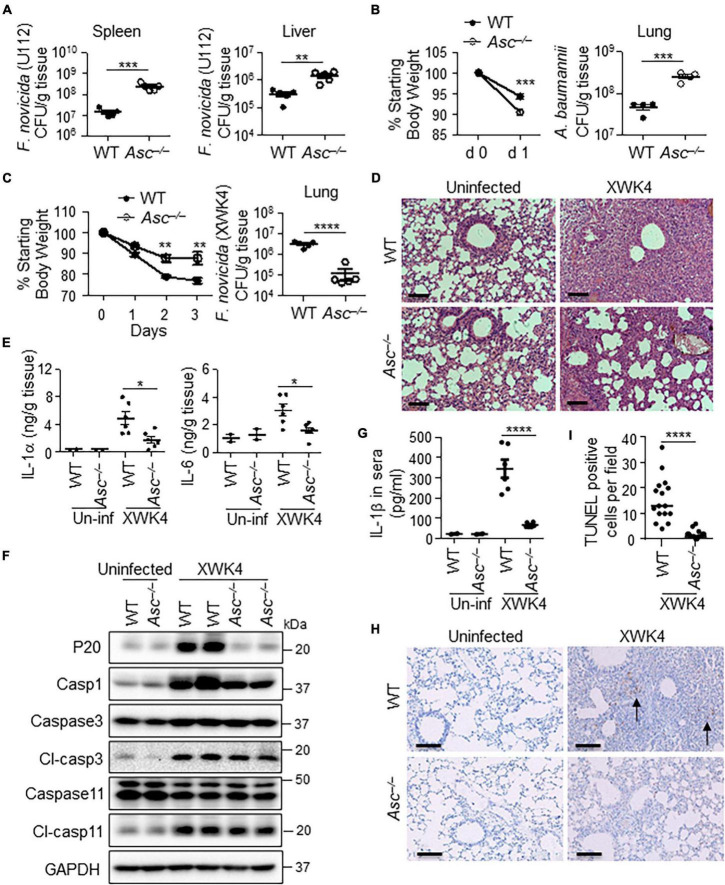
ASC activation is detrimental for host defense against XWK4 infection. **(A)** WT and *Asc^–/–^* mice were infected subcutaneously with 1.5 × 10^5^ CFU *F. novicida* (U112), and bacterial burden in the spleen and liver on day 2 after infection was measured. **(B)** WT and *Asc^–/–^* mice were infected intranasally with 5.0 × 10^8^ CFU *A. baumannii*, and body weight loss after infection and bacterial burden in the lung on day 1 after infection were analyzed. **(C)** WT and *Asc^–/–^* mice were infected intranasally with 1.0 × 10^8^ CFU *F. novicida* (XWK4), and body weight loss after infection and bacterial burden in the lung on day 3 after infection were analyzed. **(D)** H&E staining of lung sections from uninfected and XWK4-infected mice **(C)**. Scale bars: 100 μm. **(E)** ELISA analysis of IL-1α and IL-6 in the lungs from uninfected (Un-inf) and XWK4-infected mice **(C)**. **(F)** Immunoblot analysis of caspase-1, caspase-3, and caspase-11 in the lungs from uninfected and XWK4-infected mice **(C)**. **(G)** ELISA analysis of IL-1β in sera from uninfected (Un-inf) and XWK4-infected mice **(C)**. **(H)** TUNEL staining of lung sections from uninfected (Un-inf) and XWK4-infected mice **(C)**. Arrow indicates the dead cells. Scale bars: 100 μm. **(I)** Quantification analysis of TUNEL staining cells per field in the lung from XWK4-infected mice **(H)**. *p < 0.05; **p < 0.01; ***p < 0.001; ****p < 0.0001 (one-way ANOVA with multiple comparisons and two-sided Student’s *t*-test without multiple comparisons correction). Data are representative of two independent experiments.

The activation of AIM2 and NLRP3 inflammasomes typically plays positive roles in host defense against bacterial infection (Xu et al., 2019). Indeed, we confirmed that ASC-deficient mice were more susceptible to *F. novicida* U112 and *Acinetobacter baumannii* (*A. baumannii*) infection than WT mice. After subcutaneously infected with *F. novicida* U112, the bacterial burden in the spleen and liver of *Asc^–/–^* mice was much higher than that of WT mice (Figure 4A). After intranasal infection with *A. baumannii*, *Asc^–/–^* mice lost more body weight and exhibited more bacterial burden in the lung than did WT mice (Figure 4B). Surprisingly, we found that ASC deficiency resulted in increased host defense against *F. novicida* XWK4 infection. After intranasal infection with XWK4, the body weight loss and bacterial burden in the lung were significantly less in *Asc^–/–^* mice than in WT mice (Figure 4C). The less bacterial number in *Asc^–/–^* mice was associated with less inflammatory cytokine production and immune cells infiltration (Figures 4D,E). Consistently, the caspase-1 activation and IL-1β secretion in the sera induced by XWK4 infection were abolished in *Asc^–/–^* mice (Figures 4F,G). Instead, the activation of caspase-3 and caspase-11 was comparable between WT and *Asc^–/–^* mice in response to XWK4 infection (Figure 4F). In addition, Terminal deoxynucleotidyl transferase dUTP nick end labeling (TUNEL) staining revealed that the level of cell death of the lungs in XWK4-infected *Asc^–/–^* mice was less than that in WT mice (Figures 4H,I). In summary, these data indicate that mutant XWK4 triggers continuously high inflammasome activation causing excessive pathological damage and attenuated host defense.

## Discussion

Pulmonary infection with *F. novicida* WT strain is highly virulent to mice, whereas XWK4 mutant strain is avirulent *via* inducing profoundly inflammatory responses (Wang et al., 2007). Given cell death-driven inflammation in host defense mechanism, we sought to study the cell death triggered by XWK4 and its role in host defense, in particular the inflammasome activation-mediated pyroptosis. AIM2 inflammasome activation is dramatically protective for host counteract *F. novicida* infection (Fernandes-Alnemri et al., 2010). We found that mutant XWK4 induced robust inflammasome activation mediated by both AIM2 and NLRP3 receptors and the inflammasome activation was instead detrimental for bacterial clearance. Inflammasome activation typically plays positive roles in host defense against intracellular bacterial infection, and certain bacterial pathogens evolve multiple strategies for suppression of inflammasome activity and immune evasion (Brewer et al., 2019). In contrast, some bacterial pathogens are able to actively trigger inflammasome activation for promoting pathogenesis. For example, activation of NLRC4 inflammasome in alveolar macrophage triggered by *Pseudomonas aeruginosa* causes impaired bacterial clearance and is associated with increased cell death and mortality in murine model of acute pneumonia (Cohen and Prince, 2013). In addition, NLRP3 inflammasome activation trigged by *L. monocytogenes* and *Mycobacterium marinum* infection was examined as detrimental for host survival and bacterial pathogens exploited NLRP3 for exacerbation of diseases (Carlsson et al., 2010; Clark et al., 2018; Brewer et al., 2019). Our data demonstrate that the alternate function of AIM2 and NLRP3 in XWK4-triggered inflammasome assembly might result in constitutively activation of inflammasome and pathological damage.

The number of acyl chains and phosphate position in lipid A are important determinants of LPS structure to host immunostimulatory potency (Paciello et al., 2013). The smaller number of acyl chains in lipid A is associated with stronger membrane permeability and lower activity to induce proinflammatory cytokine production (Li et al., 2013). Thus, a number of bacterial pathogens have evolved lower number of acyl chains on lipid A for immune evasion and survival strategies (Steimle et al., 2016). *F. novicida* WT strain U112 synthesizes tetra-acylated lipid A lacking 4′-phosphage group and 3′-acyl chain, whereas XWK4 is penta-acylated lipid A retaining both 4′-phosphage group and 3′-acyl chain (Wang et al., 2007). Penta-acylated lipid A is more effective to facilitate NF-κB signaling through TLR4 recognition than tetra-acylated lipid A (Reife et al., 2006). The intracellular replication of XWK4 mutant was substantially reduced, although the cell entry efficiency was not affected. It was unexpected to observe that XWK4 triggered higher intensity of inflammasome activation than U112. This result suggests that the membrane permeability of XWK4 is increased that may cause more exposure of ligands for engagement with both NLRP3 and AIM2. XWK4 mutant is different from MglA mutant that is deficient in both intracellular replication and inflammasome activation due to impaired phagosome escape to the cytosol (Mariathasan et al., 2005; Brotcke et al., 2006; Jones et al., 2010). IFN-I is required for AIM2 inflammasome activation response to *F. novicida* U112 infection, because the expression of critical host factors involved in intracellular bacteriolysis is dependent on IFN-I signaling (Man et al., 2015). We found that IFN-I signaling was not essential for XWK4-triggered inflammasome activation, suggesting that the exposure of ligands engaged with NLRP3 and AIM2 might be through a different mechanism that needs to be further examined. Thus, XWK4 triggered both inflammatory genes expression and inflammasome activation, the attenuated phenotype mostly depends on the inflammatory cytokine production, while the inflammasome activation-driven inflammation and pathological damage play negative roles in host defense. Our finding will help guide the development of *Francisella* and even other bacterial vaccines to achieve maximum efficacy.

## Experimental Procedures

### Mice

*Ifnar^–/–^*, *Aim2^–/–^*, *Nlrp3^–/–^*, *Aim2^–/–^Nlrp3^–/–^*, *Raptor*^*f/f*^-CreER, and *Asc^–/–^* mice were previously described (Li Y. et al., 2018; Guo X. et al., 2020; Guo Y. et al., 2020). WT and knockout mice were kept under specific pathogen-free conditions in the Animal Resource Center at Kunming Institute of Zoology, Chinese Academy of Sciences. All animal experiments were conducted in accordance with the guidelines and were approved by the Animal Care and Use Committee, Kunming Institute of Zoology, Chinese Academy of Sciences. The animal experiment in this protocol was SMKX-2016020, with a validity period from January 2017 to January 2022.

### Bacterial Infection of Mice

The bacterial strains used in this study included *F. novicida* WT strain U112, mutant XWK4, and *A. baumannii* that were growing as previously described (Wang et al., 2007; Li Y. et al., 2018).

Eight- to 10-week-old and gender-matched WT and knockout mice were infected subcutaneously with *F. novicida* U112 (1.5 × 10^5^ CFUs per mouse), intranasally with *F. novicida* U112 (1.0 × 10^4^ CFUs per mouse), XWK4 (1.0 × 10^5^ or 1.0 × 10^8^ CFUs per mouse), or *A. baumannii* (5.0 × 10^8^ CFUs per mouse) as indicated. Mice were weighed and monitored daily over time. Mice were euthanized at days as indicated after infection, and the liver, spleen, and lung were harvested to determine the bacterial burden.

### Preparation of Bone Marrow-Derived Macrophage and Bacterial Infection

To generate BMDMs, bone marrow (BM) cells were cultured in L929 cell-conditioned DMEM/F-12 supplemented with 10% FBS, 1% non-essential amino acids, and 1% penicillin–streptomycin for 5 days as previously described. WT and knockout BMDMs were infected with bacterial pathogens for indicated times as previously described (Guo Y. et al., 2020). The uninfected and infected BMDM cells were lysed for RNA and protein analysis.

### Transmission Electron Microscopy

U112- and XWK4-infected BMDMs were fixed in 2% paraformaldehyde and 2.5% glutaraldehyde in 0.1 M cacodylate buffer (pH 7.4) for 1 h at 37°C. Cells were embedded and sectioned for transmission electron microscopy by the Imaging Core Facility of Kunming Institute of Zoology.

### Bacterial Killing Assay

Bone marrow-derived macrophages were infected with *F. novicida* U112 and XWK4 with a multiplicity of infection (MOI) of 20 for 2 h, washed, and added with gentamicin (50 μg/ml) to kill extracellular bacteria. After 1 h, cells were washed twice and cultured in fresh media. BMDMs were lysed in PBS at indicated times post-infection, serially diluted, plated onto TSB agar plates, and incubated overnight for CFU enumeration.

### Preparation of Lung Sample for H&E and Terminal Deoxynucleotidyl Transferase dUTP Nick end Labeling Staining and Immunoblot Analysis

The superior lobes of the right lungs were fixed in 10% formalin, and 5-μm sections were stained with H&E and examined. TUNEL staining was performed with the TUNEL assay kit (G1507) according to the manufacturer’s instructions (Servicebio).

Uninfected and infected lung tissues were homogenized in RIPA buffer with protease and phosphatase inhibitors for immunoblot analysis. Protein concentration was determined using bicinchoninic acid assay (BCA) kit (Pierce) according to the manufacturer’s instructions.

### Immunoblot Analysis and Antibodies

Samples were separated by 12% SDS-PAGE, followed by electrophoretic transfer to polyvinylidene fluoride membranes and blocking and incubating membranes with primary antibodies. The following primary antibodies were used: anti-caspase-1 (AG-20B-0042; AdipoGen), anti-caspase-3 (9661 and 9662; CST), anti-caspase-11 (NB120-10454; Novus Biologicals), anti-AIM2 (13095; CST), anti-NLRP3 (AG-20B-0014; AdipoGen), anti-TFEB (A303-673A; Bethyl Laboratories), anti-LC3 (catalog NB600-1384; Novus Biologicals), and anti-GAPDH (5174; CST). HRP-labeled anti-rabbit or anti-mouse antibodies (Cell Signaling Technology) were used as secondary antibodies.

### Real-Time qRT-PCR

Total RNA was isolated from cells and tissues by using TRIzol reagent (Invitrogen). cDNA was reverse transcribed by using M-MLV reverse transcriptase (Promega). Real-time qRT-PCR was performed on the Bio-Rad CFX-96 Touch Real-Time Detection System. Primer sequences are listed in Supplementary Table 1.

### LDH Release Assay

Cell culture supernatants were collected at the indicated times, and lactate dehydrogenase activity was measured by using the Promega cytotoxicity kit according to the manufacturer’s protocols.

### ELISA

The *in vivo* and *in vitro* samples were analyzed for cytokine release by using ELISA MAX^TM^ Standard (mouse IL-1β, 432601; mouse IL-6, 431301; mouse IL-1α, 433401; BioLegend) as per the manufacturer’s instructions.

### Statistical Analyses

Data are given as mean ± standard error of the mean. Statistical analyses were performed by using one-way ANOVA with multiple comparisons, two-tailed Student’s *t*-test, and log-rank tests. *P*-values ≤ 0.05 were considered significant.

## Conclusion

Inflammasome activation contributes to the proinflammatory cytokine maturation and pyroptotic cell death during bacterial infection, and the cell death-driven inflammation is critical for host defense against bacterial pathogens. Here, we find that *Francisella novicida* mutant XWK4 triggers inflammatory cytokine expression and robust inflammasome activation that is dependent on both NLRP3 and AIM2. However, the inflammasome activation is detrimental for host defense against XWK4 infection due to excessive pathological damage. Our study provides novel insights into the role of inflammasome activation in host defense against bacterial infection.

## Data Availability Statement

The datasets presented in this study can be found in online repositories. The names of the repository/repositories and accession number(s) can be found in the article/Supplementary Material.

## Ethics Statement

The animal study was reviewed and approved by the Animal Care and Use Committee, Kunming Institute of Zoology, Chinese Academy of Sciences. Written informed consent was obtained from the owners for the participation of their animals in this study.

## Author Contributions

XQ designed the study and wrote the manuscript. YG, RM, QX, XC, TX, XW, YD, and XQ performed the experiments and analyzed the data. All authors contributed to the article and approved the submitted version.

## Conflict of Interest

The authors declare that the research was conducted in the absence of any commercial or financial relationships that could be construed as a potential conflict of interest.

## Publisher’s Note

All claims expressed in this article are solely those of the authors and do not necessarily represent those of their affiliated organizations, or those of the publisher, the editors and the reviewers. Any product that may be evaluated in this article, or claim that may be made by its manufacturer, is not guaranteed or endorsed by the publisher.

## Supplementary Material

Supplementary Figure 1 XWK4 dramatically reduces virulence. **(A,B)** WT mice were infected intranasally with *F. novicida* U112 (1 × 10^4^ CFU) and XWK4 (1 × 10^5^ CFU), and survival **(A)** and body weight loss **(B)** after infection were analyzed. **(C,D)** *Aim2^–/–^* mice were infected intranasally with *F. novicida* U112 (1 × 10^4^ CFU) and XWK4 (1 × 10^5^ CFU), and survival **(C)** and body weight loss **(D)** after infection were analyzed. **(E,F)** *Ifnar^–/–^* mice were infected intranasally with *F. novicida* U112 (1 × 10^4^ CFU) and XWK4 (1 × 10^5^ CFU), and survival **(E)** and body weight loss **(F)** after infection were analyzed. ^∗∗^*P* < 0.01; ^****^*P* < 0.0001 (one-way ANOVA with multiple comparisons). Data are representative of 2 independent experiments.

Supplementary Figure 2 mTORC1 signaling is dispensable for XWK4-triggered inflammasome activation. **(A)** CreER-expressed *Raptor*^+/+^ and *Raptor*^*f/f*^ BMDMs were treated with 4-OHT (100 nM) for 5 days to induce Raptor gene deletion. Immunoblot analysis of Caspase-1 in WT and *Raptor^–/–^* BMDMs with and without infection of U112 and XWK4 (200 MOI) for indicated times. **(B)** Immunoblot analysis of Caspase-1 in untreated and rapamycin-treated BMDMs (500 nM) with and without infection of U112 and XWK4 (200 MOI) for 20 h. Data are representative of three independent experiments.
