# Supplementary figures and images for: Francisella novicida Mutant XWK4 Triggers Robust Inflammasome Activation Favoring Infection
Source: Front Cell Dev Biol. 2021 Nov 18;9:743335. doi: 10.3389/fcell.2021.743335 (PMC8637620; doi:10.3389/fcell.2021.743335)

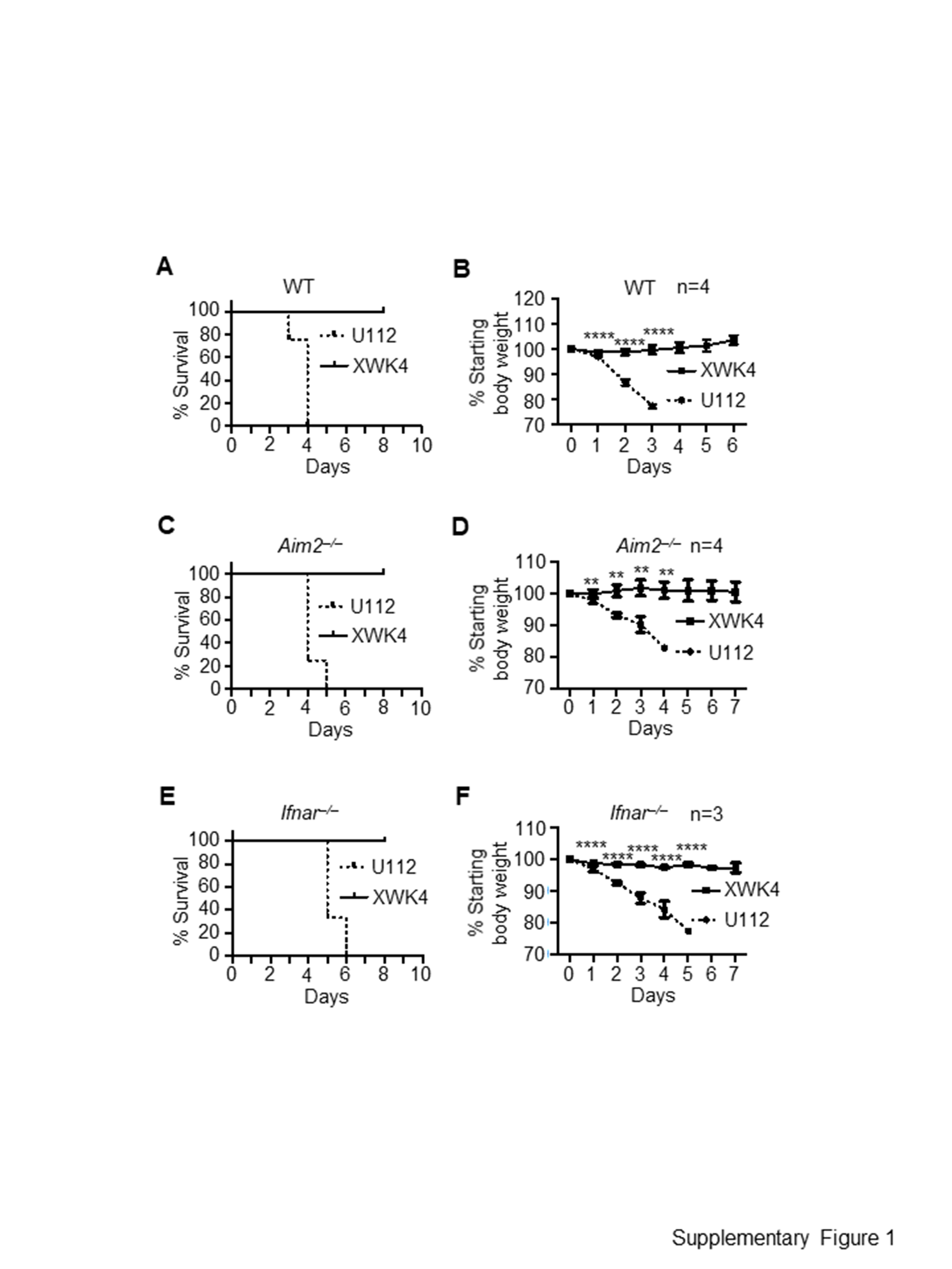

Supplement: Supplementary Figure 1 — XWK4 dramatically reduces virulence. (A,B) WT mice were infected intranasally with F. novicida U112 (1 × 10^4^ CFU) and XWK4 (1 × 10^5^ CFU), and survival (A) and body weight loss (B) after infection were analyzed. (C,D) Aim2–/– mice were infected intranasally with F. novicida U112 (1 × 10^4^ CFU) and XWK4 (1 × 10^5^ CFU), and survival (C) and body weight loss (D) after infection were analyzed. (E,F) Ifnar–/– mice were infected intranasally with F. novicida U112 (1 × 10^4^ CFU) and XWK4 (1 × 10^5^ CFU), and survival (E) and body weight loss (F) after infection were analyzed. ∗∗P < 0.01; ****P < 0.0001 (one-way ANOVA with multiple comparisons). Data are representative of 2 independent experiments. [file Image_1.TIF]

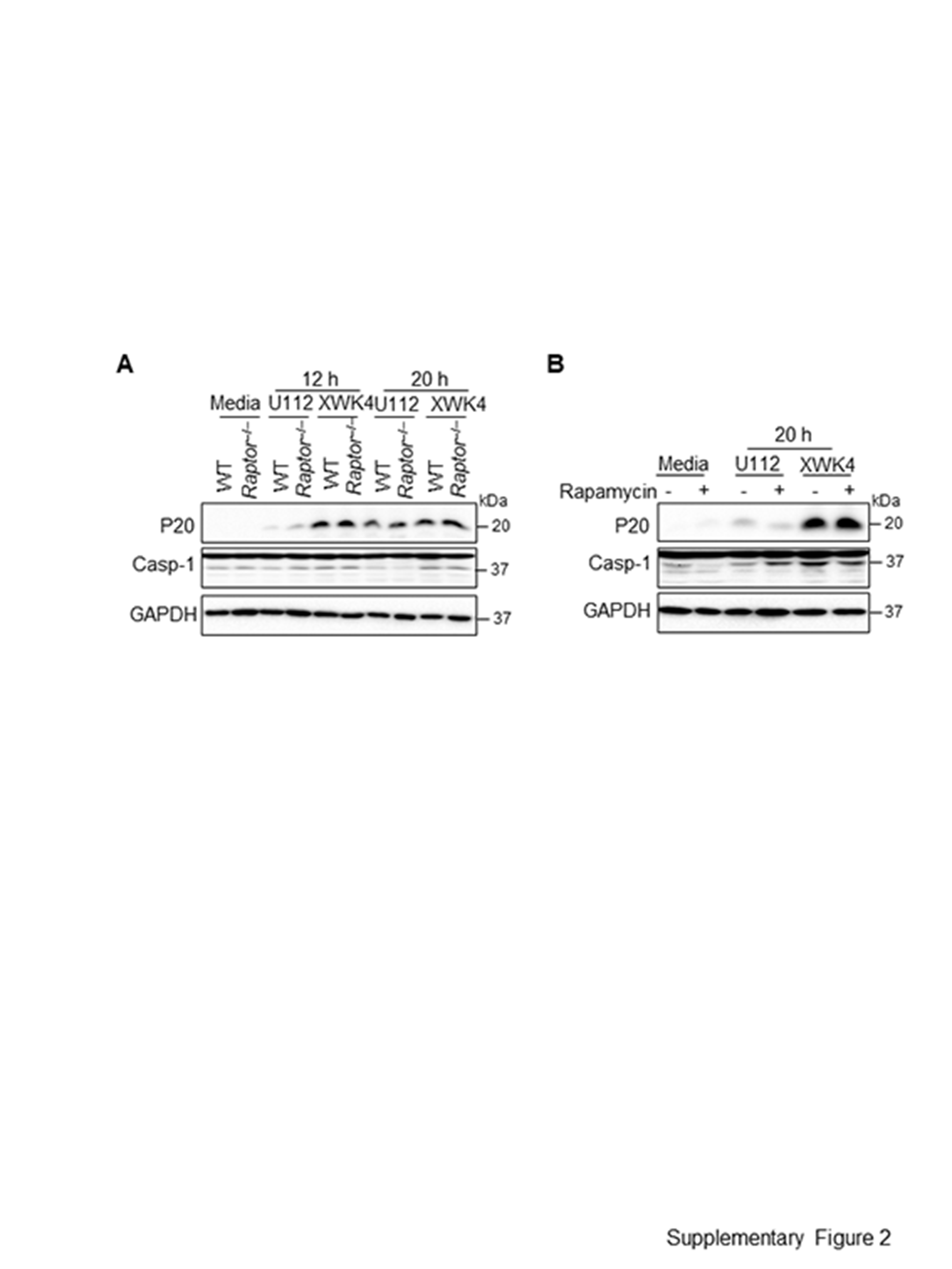

Supplement: Supplementary Figure 2 — mTORC1 signaling is dispensable for XWK4-triggered inflammasome activation. (A) CreER-expressed Raptor+/+ and Raptorf/f BMDMs were treated with 4-OHT (100 nM) for 5 days to induce Raptor gene deletion. Immunoblot analysis of Caspase-1 in WT and Raptor–/– BMDMs with and without infection of U112 and XWK4 (200 MOI) for indicated times. (B) Immunoblot analysis of Caspase-1 in untreated and rapamycin-treated BMDMs (500 nM) with and without infection of U112 and XWK4 (200 MOI) for 20 h. Data are representative of three independent experiments. [file Image_2.TIF]
